# Broad spectrum resistance in *Helicobacter pylori* isolated from gastric biopsies of patients with dyspepsia in Cameroon and efflux-mediated multiresistance detection in MDR isolates

**DOI:** 10.1186/s12879-019-4536-8

**Published:** 2019-10-22

**Authors:** Laure Brigitte Kouitcheu Mabeku, Bertrand Eyoum Bille, Cromwell Tepap Zemnou, Lionel Danny Tali Nguefack, Hubert Leundji

**Affiliations:** 1 Microbiology and Pharmacology Laboratory, Department of Biochemistry, Faculty of Science, University of Dschang, P. O. Box, 67 Dschang, Cameroon; 2 Gastroenterology Department, Laquintinie Hospital of Douala, P. O. Box, 4035 Douala, Cameroon

**Keywords:** *Helicobacter pylori*, Resistance prevalence, Antibiotics, Efflux pump, Cameroon

## Abstract

**Background:**

Antibiotic resistance is a leading cause of treatment failure in *Helicobacter pylori* infection. In Africa, there are very little data concerning the susceptibility of *Helicobacter pylori* isolates to antibiotics. The purpose of this study was to evaluate the resistance prevalence of *Helicobacter pylori* strains circulating in Cameroon, and to assess overexpression of efflux pump as a possible multi-drug resistance mechanisms.

**Methods:**

A total of 140 *H. pylori* isolates were recovered from gastric biopsies of dyspeptic patients in two reference hospitals in Cameroon and analyzed for their antimicrobial susceptibility to amoxicillin, co-amoxiclav, ampicillin, penicillin, imipenem, metronidazole, rifabutin, erythromycin, clarithromycin, azithromycin, levofloxacin, ciprofloxacin, norfloxacin, tetracycline, doxycycline and minocycline. Antibiotic sensitivity was tested by disk diffusion method. Phe-Arg-naphthylamide (PAßN) was used as efflux pump inhibitor. INT broth microdilution method in supplemented Brain Heart Infusion broth was used to determine the MIC of ampicillin, amoxicillin, metronidazole, erythromycin, clarithromycin and doxycycline in the absence and the presence of PAßN against 32 selected MDR isolates.

**Results:**

Overall *H. pylori* resistance rate was 100% to ampicillin, penicillin and co-amoxiclav; 97.14% to amoxicillin, 97.85% to metronidazole, 47.85% to erythromycin, 13.57% to clarithromycin; 5, 2.86 and 0.71% to doxycycline, tetracycline and minocycline respectively. No resistance to azithromycin, rifabutin, imipenem, ciprofloxacin, norfloxacin and levofloxacin was detected among *H. pylori* isolates. Seventy percent (70%) of the tested isolates elicited a multiple drugs resistance pattern; 42.57% double, 15.71% triple and 5.71% quadruple drugs resistance. Metronidazole and amoxicillin were more concerned with double resistance pattern (86.76%). The spectrum of activity recorded with metronidazole, doxycycline, clarithromycin and erythromycin ranged from 0 to 100% in the absence to the presence of PAßN against the tested MDR isolates. An 8 to 128-fold increase in potency was also noticed with these antibiotics in the presence of PAßN.

**Conclusion:**

With regard to the high resistance rate to both amoxicillin and metronidazole, these drugs should be avoided as components of triple therapy in our milieu. In contrast, ciprofloxacin, norfloxacin, levofloxacin and tetracyclines could be used to achieve a better eradication rate and to reduce the risk of selection of *H. pylori* resistant strains.

## Background

*Helicobacter pylori (H. pylori)* is the only known pathogen that inhabits the gastric mucosa of almost half of the world’s population [1, 2]. This bacterium can remain asymptomatic or can cause several gastrointestinal diseases, ranging in severity from superficial and chronic gastritis to duodenal ulceration and gastric adenocarcinoma [3].

In general, combined therapy is used to eradicate *H. pylori* infection [4]. Triple therapy, including two antibiotics, amoxicillin and clarithromycin, and a proton pump inhibitor given for a week has been recommended as the treatment of choice at several consensus conferences [5, 6]. However, the efficiency of this standard regimen has decreased over the past decades, with the overall success rate of 74.6% in an intention-to-treat analysis and 82% in a per-protocol analysis [7]. In fact, the main reason for failure was found to be *H pylori* resistance to one of the antibiotics used (that is, clarithromycin) [8, 9]. Other treatments have also been proposed, including metronidazole, as well as tetracycline, fluoroquinolones, and rifamycins for which resistance has become an emerging issue [10, 11]. Resistance of *H. pylori* to the limited range of antibiotics that have efficacy in its treatment can severely affect attempts to eradicate this bacteria. Therefore, susceptibility testing of *H. pylori* is important for the eradication of this organism. An additional advantage of susceptibility testing is that it may reduce the risk of *H. pylori* resistance [12]. As a result, the choice of regimens for patients should be based on knowledge of local resistance patterns and antibiotic used [13].

Bacterial resistance to antimicrobial agents may be caused by overexpression of multi-drug resistance (MDR) efflux pumps [14, 15]**.** Active efflux was first described in 1980, as a causative mechanism of resistance to tetracyclines [16]**.** It has subsequently been found to be a widespread mechanism conferring to both Gram-positive and Gram-negative organisms the capacity to expel antibiotics from all the major structural classes [15, 17]. Among Gram-negative bacteria, many of these MDR efflux pumps belong to the resistance-nodulation-cell division (RND) type family of tripartite efflux pumps [18]**.** MDR in selected Gram-negative bacteria has been shown to be reversible by using compounds like Phe-Arg-naphthylamide (PAβN) or other small N-heterocyclic organic compounds thought to inhibit RND type efflux pump activity through unknown mechanisms [19–22].

*H. pylori* infection seems to be common in Cameroon: a study carried out in 2004 had demonstrated a prevalence of 92.2% among apparently healthy children in the Buea and Limbe health districts of Cameroon [23]; a hospital-based survey conducted in 2013 has revealed an overall prevalence of 72.5% (124/171) in Yaoundé, center region of Cameroon [24]. Still in the same region in 2015, a prevalence of 79.3% of the pathogen was documented among children and adolescents from the age range of 6 to 18 years old with peptic ulcer disease [25]. In 2016, Kouitcheu et al. [26] found a seroprevalence of 64.34% among 205 patients aged 35 years and older with symptoms of dyspepsia or other symptoms referable to the proximal alimentary tract in the littoral region of Cameroon. Even though *H. pylori* infection is common in Cameroon, there is no regional surveillance programs that monitor the evolution of *H. pylori* resistance in order to allow timely adaptation of the treatment regimens in the country. The present study is therefore aimed at evaluating the susceptibility of clinical isolates of *H. pylori* circulating in Cameroon to different antibiotics used in the treatment of this infection, in a bid to identify potential optimal therapeutic regimen for this infection adapted to Cameroon. As a causative mechanism of resistance, the spectrum of action of these antibiotics regarding the role of efflux pumps in their activity was also investigated by using multi drug resistant strains selected and Phe-Arg-naphthylamide (PAßN) a previously described efflux pump inhibitor.

## Methods

### Selection of subjects

This was a cross sectional study carried out at the gastroenterology Department of Laquintinie Hospital and General Hospital of Douala-Cameroon, from April 2013 to June 2015. All patients aged 15 to 90 years old, either sex, attending the Gastroenterology Department at the selected health centers with gastritis, gastric and duodenal ulcer undergoing endoscopy were recruited. Exclusion criteria were, (1) Non-cooperative patients who refused to give their consent or to participate to the study; (2) patients with a history of *H. pylori* eradication treatment; (3) patients with a history of antibiotics consumption within the last fourth weeks. Gastric biopsies were collected from the enrolled patients, ground and about two drops of homogenates were inoculated into supplemented Columbia Agar (Columbia Agar + 5% (v/v) lacked horse blood and 1% (v/v) Vitox (CD-Vitox)). The inoculated culture media were incubated at 37 °C under microaerophilic conditions (CampyGen gas pack) for 7 days. Isolates that exhibited Gram negative curved rods on Gram stain reaction and were positive for catalase, oxidase and urease tests were considered as *H. pylori* [27, 28]. Confirmed isolates were suspended in eppendorf tubes containing Brain Heart Infusion broth supplemented with 5% horse serum (BHI-serum) with 30% glycerol and stored at − 80 °C until future use. The study was approved by both local ethical committee (Approval N^0^ 425/AR/MINSANTE/HLD/SCM/CR) and the national institutional Review Board, the National Ethical Committee on human health research in Cameroon (ethical clearance N^0^ 2014/03/425/L/CNESRH/SP).A standard control strain NCTC 11638 was also used to confirm the identification and for antimicrobial assays.

### Bacterial strains

One hundred and forty (140) *H. pylori* isolates were recovered from gastric biopsies of eligible patients and subjected to antimicrobial assays in this study. All isolates were removed from storage at − 80 °C and subcultured on supplemented Columbia Agar (Columbia Agar + 5% (v/v) lacked horse blood and 1% (v/v) Vitox (CD-Vitox)). Subcultures were incubated at 37 °C under microaerophilic conditions (CampyGen gas pack) for 3 days and for two passages to ensure reliable growth. Gram staining, rapid urease and the catalase/oxydase tests were performed to confirm the identification [28, 29].

### Antibiotic susceptibility test

Antibiotic susceptibility studies were performed by disc diffusion (Kirby–Bauer) method according to Clinical Laboratory Standards Institute (CLSI, 2015) [29]. The following antibiotic disks were tested: amoxicillin (10 μg), co-amoxiclav (30 μg), ampicillin (10 μg), penicillin (10 U), imipenem (10 μg), metronidazole (5 μg), rifabutin (5 μg), erythromycin (15 μg), clarithromycin (15 μg), azithromycin (15 μg), levofloxacin (5 μg), ciprofloxacin (30 μg), norfloxacin (30 μg), tetracycline (30 μg), doxycycline (30 μg) and minocycline (30 μg) (Biomaxima). *H. pylori* inocula prepared at McFarlands turbidity standard 3 (6 × 10 ^8^ CFU/ml) by suspending 48-h colonies in 2 ml-sterile normal saline was used to seed each prepared and dried supplemented Columbia agar plate. The discs were arranged and firmly pressed on the agar surface of each seeded plate. The plates were incubated under microaerophilic conditions at 37 °C for 48 to 72 h. The microaerophilic conditions were generated using CampyGen in an air-tight anaerobic jar (Oxoid). Zone sizes were measured and the isolates were classified as sensitive or resistant according to the interpretative criteria for CLSI guidelines (2012) and CASFM (2017) [30, 31]. The isolate exhibited a multidrug resistance phenotype were also noticed. The experiment was performed in triplicate and the mean zone diameters of inhibition recorded for each antibiotic.

### Assessment of MDR reversal activity in MDR *H. pylori* isolates: MIC determination of antibiotics alone and in combination with efflux pump inhibitor

In order to determine a possible mechanisms involved in resistance pattern of the tested clinical isolate against antibiotics used, overexpression of MDR efflux pumps in the isolated *H. pylori* strains was investigated using Phe-Arg-naphthylamide (PAßN). Thirty two (32) isolates which elicited triple or quadruple drug-multi resistance pattern to the tested antibiotics according to the above test were selected. Selected MDR isolates were tested for their susceptibilities to antibiotics amoxicillin (AMO), ampicillin (AMP), clarithromycin (CLR), erythromycin (ERY), metronidazole (MET), tetracycline (TET) and ciprofloxacin (CIP) alone and then in the presence of PAßN at a final concentration of 20 μg/ml as described previously [32]. The MICs were carried out by the INT broth microdilution method [33, 34] using Brain Heart Infusion broth supplemented with 5% horse serum (BHI-serum) and various concentrations of antibiotics. Two fold dilutions of each selected antibiotics were prepared in the test wells in BHI -serum. The ranges of antibiotic concentrations that were used in this study were as follows: amoxicillin (AMO, 0.03125–128 μg/ml), ampicillin (AMP, 0.03125–128 μg/ml), Clarithromycin (CLR, 0.0625–16 μg/ml), erythromycin (ERY, 0.125–32 μg/ml), metronidazole (MET, 0.5–128 μg/ml) and doxycycline (DOX, 0.0625–32 μg/ml). All antimicrobial agents were purchased from Sigma-Aldrich, Germany. The selected MDR *H. pylori* isolates were removed from freezer, thawed and subcultured on supplemented Columbia Agar without antibiotics and incubated under microaerophilic conditions at 37 °C for 3 days. After a lawn of growth appeared, MDR bacterial colonies were suspended in sterile saline at a density equivalent to 3 McFarland’s standard. One hundred microliter of each prepared inoculum was added to 100 μl of the antibiotic-containing culture medium or antibiotic+PAßN-containing culture medium. Control wells were prepared with culture medium and MDR bacterial suspension, and broth only. The plates were covered with a sterile plate sealer; the contents of the wells were mixed with a shaker and incubated for 3 days at 37∘C under microaerophilic conditions. After incubation, 40 *µ*l of 0.2 mg/ml INT was added per well and incubated at 37∘C for 30 min. Living bacteria reduced the yellow dye to pink. The MIC value of antibiotic defined as its lowest concentration which completely inhibited visible bacterial growth at 37 °C after 72 h was determined in the presence and in the absence of efflux pump inhibitor. Each MIC was determined in triplicate and the mean values were recorded. Then, the fractional inhibitory concentration (FIC) of each combination was calculated as the ratio of MIC of antibiotic with efflux pump inhibitor versus MIC of antibiotic alone**.** Resistance was defined in accordance with the European Committee on Antimicrobial Susceptibility Testing (EUCAST) guidelines [35]: amoxicillin, ampicillin (MIC > 0.125 μg/ml), clarithromycin (MIC > 0.5 μg/ml), erythromycin (MIC > 1 μg/ml), tetracycline, doxycycline (MIC > 1 μg/ml); metronidazole (MIC > 4 μg/ml) [36].

## Results

### Resistance profile of isolated *H. pylori* strains to antibiotics

**Fig. 1:**
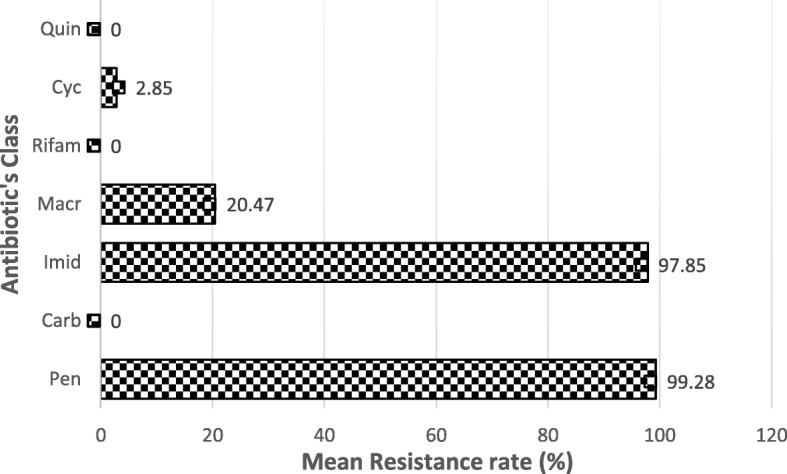
Resistance profile of *H. pylori* clinical isolates to class of antibiotics tested. Penicillin: Pen, Carbopenems: Carb, Imidazole: Imid, Macrolides: Macr, Rifamycines: Rifam, Tetracyclines: Cyc, Quinolones: Quin

In this study, the susceptibility of 140 *H. pylori* clinical isolates was evaluated against 16 antibiotics belonged to six class and they were classified according to their resistance profile to the tested drugs (Tables 1 and 2, Fig. 1). None of the tested strains elicited a 100% susceptibility to the overall antibiotics used. However, they did not showed any resistance to rifabutin, imipenem, azithromycin, ciprofloxacin, norfloxacin and levofloxacin (Table 2). Resistance rates recorded against the tested antibiotics were as follows: highest resistance rate (100 to 97.85%) to metronidazole and penicillin group (ampicillin, penicillin, co-amoxiclav, amoxicillin); 47.85 and 13.57% to erythromycin and clarithromycin respectively. Antibiotic from tetracyclines (tetracycline: 2.86%, doxycycline: 5% and minocycline: 0.71%) were slightly affected by resistance. Since there was a high disparity between the resistance rate of imipenem (0%) and that of the other β lactam antibiotics tested (ampicillin: 100%, penicillin: 100%, co-amoxiclav: 100% and amoxicillin: 97.14%), this class were divided in penicillin and carbopemems subgroups, and the resistance prevalence of this two subgroups were not joined in statistical analysis. So, the peak of resistance was detected with metronidazole (imidazoles class) and penicillin’s subgroup with respective resistance rate of 99.28 and 97.85%, followed by macrolides with 20.47%. However, none of the tested strains were resistance to azithromycin one of the three macrolides tested (Fig. 1). Rifabutin, the only antibiotic of the rifamycine class used and all the fluoroquinolone tested showed a 100% activity against the tested isolates.

**Table 1:** Screening of antibiotics for anti-*H. pylori* activity using disc diffusion method

Antibiotics	Breakpoint	Mean zone diameter (mm)	Inhibition diameter range (mm)
Amoxicillin (AMO)	≥21 < 16	6.21 ± 1.35	6–12
Co-Amoxiclav (AMC)	≥23 < 16	6.59 ± 3.02	6–28
Penicillin (PEN)	≥23 < 16	6.125 ± 0.5	6–8
Imipenem (IMP)	≥29 < 18	6.25 ± 0.61	6–8
Metronidazole (MET)	≥24 < 17	22 ± 1.63	20–24
Clarithromycin (CLR)	≥ND < 21	6.49 ± 2.43	6–22
Azithromycin (AZT)	≥22 < 16	22.91 ± 4.25	10–30
Erythromycin (ERY)	≥22 < 16	29.89 ± 6.25	18–40
Rifabutin (RIF)	≥22 < 17	17.59 ± 6.58	6–30
Tetracycline (TET)	ND < 17	20.61 ± 1.93	18–32
Doxycycline (DOX)	≥19 < 17	20.83 ± 3.11	12–30
Minocycline (MIN)	≥19 < 17	22.55 ± 4.53	16–34
Levofloxacin (LEV)	≥19 < 17	23.08 ± 2.29	18–28
Ciprofloxacin (CIP)	≥25 < 20	31.40 ± 3.99	22–40
Norfloxacin (NOR)	≥25 < 12	31.20 ± 3.42	20–40
Ampicillin (AMP)	≥25 < 20	28.38 ± 2.27	22–32

Data are mean ± SD of 140 determinations for each antibiotic

**Table 2:** Resistance of *Helicobacter pylori* clinical isolates to antibiotics

Antibiotics	Resistance No (%)
Ampicillin (AMP)	140 (100)
Amoxicillin (AMO)	136 (97.14)
Co-Amoxiclav (AMC)	140 (100)
Penicillin (PEN)	140 (100)
Imipenem (IMP)	0(0)
Metronidazole (MET)	137 (97.85)
Clarithromycin (CLR)	19 (13.57)
Azithromycin (AZT)	0(0)
Erythromycin (ERY)	67(47.85)
Rifabutin (RIF)	0(0)
Tetracycline (TET)	4(2.86)
Doxycycline (DOX)	7(5)
Minocycline (MIN)	1(0.71)
Levofloxacin (LEV)	0(0)
Ciprofloxacin (CIP)	0(0)
Norfloxacin (NOR)	0(0)

No: Number of resistant isolate to each antibiotic

### Multiple resistance pattern of isolated *H. pylori* strains

Out of the 140 *H. pylori* isolates tested, 98 elicited a multiple resistance pattern, given the overall multidrug resistance rate of 70% (98/140). The incriminated antibiotics classes were imidazole, penicillin, macrolides and tetracyclines. Table 3 summarizes the multi-drug resistant pattern of tested *H. pylori* isolates; 68 (48.57%) elicited double, 22 (15.71%) triple and 8 (5.71%) quadruple drugs resistance. Double resistance were more frequent with metronidazole and amoxicillin (MET^R^/AMO^R^) which represented 86.76% (59/68) of this type of multi-drugs resistance pattern, followed by MET^R^/CLR^R^, MET^R^/TET^R^ and CLR^R^/AMO^R^. As regards triple drug resistance, metronidazole, clarithromycin and amoxicillin (MET^R^/CLR^R^/AMO^R^) were more concerned and represented 95.45% (21/22) of this type of multidrug resistance pattern. Quadruple drugs resistance was the maximum and the less multi-drugs resistance pattern detected and metronidazole, tetracycline, clarithromycin and amoxicillin were combined.

**Table 3:** Multi-drugs resistance pattern of *Helicobacter pylori* clinical isolates to antibiotic

Multi-resistance pattern	Double drugs resistance				Triple drugs resistance		Quadruple drugs resistance	Overall multi-drugs resistance
	MET^R^ AMO ^R^	MET^R^ CLR^R^	CLR^R^ AMO^R^	MET^R^/TET^R^	MET^R^ CLR^R^ AMO ^R^	MET^R^TET^R^ AMO ^R^	MET^R^TET^R^ CLR^R^AMO ^R^	
Number (%)	59 (42.14)	6 (4.28)	1 (0.71)	2 (1.42)	21 (15)	1 (0.71)	8 (5.71)	
Total (%)	68 (48.57)				22 (15.71)		8(5.71)	98(70)

### Efflux-mediated antimicrobial resistance

As possible mechanisms involved in resistance pattern of the tested clinical isolates against antibiotics used, overexpression of MDR efflux pumps in the isolated *H. pylori* strains was investigated using Phe-Arg-naphthylamide (PAßN). The susceptibility to antibiotics of selected MDR isolates (32) was evaluated in the presence and absence of the PAßN. The selected antibiotics were ampicillin, amoxicillin, metronidazole, erythromycin, clarithromycin and doxycycline to which the tested isolate displayed a resistance prevalence of 100, 97.14, 97.85, 47.85, 13.57 and 5% respectively. Table 4 summarizes the susceptibility of the selected MDR *H. pylori* isolate to antibiotics in the absence and presence of PAβN. The activity spectrum of amoxicillin and ampicillin was not affected in the presence of the efflux pumps inhibitor. However, our results showed that this inhibitor (PAßN) have improved the activity of 67% (4/6) of the antibiotics tested (that is metronidazole, doxycycline, clarithromycin and erythromycin). The spectrum of activity recorded with metronidazole, doxycycline, clarithromycin and erythromycin against the tested MDR *H. pylori* strains ranged in the absence to the presence of the inhibitor from 0 to 34.37%; from 40 to 100%; from 0 to 100% and from 6.90 to 100% respectively (Table 4).

**Table 4:** MIC value of antibiotics (μg/ml) in absence and in presence of the efflux pump inhibitor (PAßN)

*H. pylori* clinical isolate	Antibiotics																	
	MET			CLR			ERY			AMP			AMO			DOX		
	A	P	F	A	P	F	A	P	F	A	P	F	A	P	F	A	P	F
HP	128	4	31.2	16	0.25	15.2	8	<0.2	31.2	≠	≠	/	≠	≠	/	2	<0.2	125
HP002	128	4	31.2	2	<0.2	125	2	<0.2	125	≠	≠	/	≠	≠	/	–	–	–
HP0094	128	8	62.5	–	–	–	4	<0.2	62.5	≠	≠	/	≠	≠	/	2	<0.2	125
HP0072	128	4	125	–	–	–	2	0.5	250	≠	≠	/	≠	≠	/	–	–	–
HP0099	128	16	125	–	–	–	2	0.25	125	≠	≠	/	≠	≠	/	–	–	–
HP0095	128	2	15.6	4	<0.2	62.5	8	0.25	31.2	≠	≠	/	≠	≠	/	–	–	–
HP00115	128	8	62.5	1	<0.2	250	2	<0.2	125	≠	≠	/	≠	≠	/	–	–	–
HP00117	128	8	62.5	8	<0.2	31.2	8	0.25	31.2	≠	≠	/	≠	≠	/	–	–	–
HP00105	128	4	31.2	–	–	–	8	0.25	31.2	≠	≠	/	≠	≠	/	1	<0.2	250
HP0060	128	16	125	–	–	–	1	0.25	250	≠	≠	/	≠	≠	/	–	–	–
HP0063	128	8	62.5	–	–	–	8	<0.2	31.2	≠	≠	/	–	–	–	–	–	–
HP0050	128	8	62.5	–	–	–	4	<0.2	62.5	≠	≠	/	≠	≠	/	–	–	–
HP0048	128	16	125	–	–	–	4	<0.2	62.5	≠	≠	/	≠	≠	/	–	–	–
HP0069	128	4	31.2	–	–	–	2	0.5	125	≠	≠	/	≠	≠	/	–	–	–
HP0070	128	4	31.2	–	–	–	4	<0.2	62.5	≠	≠	/	≠	≠	/	0.5	<0.2	500
HP0055	128	4	31.2	–	–	–	8	1	125	≠	≠	/	≠	≠	/	2	<0.25	125
HP00102HD	128	8	62.5	–	–	–	4	0.25	31.2	≠	≠	/	≠	≠	/	–	–	–
HP00132	128	16	125	–	–	–	1	0.25	250	≠	≠	/	≠	≠	1	–	–	–
HP00131	≠	8	62.5	–	–	–	8	0.25	31.2	≠	≠	/	≠	≠	/	–	–	–
HP00133	128	16	125	–	–	–	–			≠	≠	/	≠	≠	/	–	–	–
HP0045HGD	128	16	125	–	–	–	8	<0.2	31.25	≠	≠	/	≠	≠	/	–	–	–
HP0098HGD	128	8	62.5	–	–	–	–			≠	≠	/	≠	≠	1	–	–	–
HP00100HD	128	8	62.5	–	–	–	4	0.25	62.5	≠	≠	/	≠	≠	/	–	–	–
HP0064	≠	1	7.8	–	–	–	8	<0.2	31.2	≠	≠	/	≠	≠	/	–	–	–
HP0065	128	8	62.5	–	–	–	–			≠	≠	/	≠	≠	/	–	–	–
HP0066	128	16	125	–	–	–	8	<0.2	31.2	≠	≠	/	≠	≠	/	–	–	–
HP0063HGD	≠	8	62.5	–	–	–	4	0.25	62.5	≠	≠	/	≠	≠	/	–	–	–
HP0044HGD	128	16	125	–	–	–	4	0.25	62.5	≠	≠	/	–	–		–	–	–
HP0046HGD	128	16	125	–	–	–	4	0.25	62.5	≠	≠	/	≠	≠	/	–	–	–
HP0036	128	4	31.25	–	–	–	8	0.25	31.2	≠	≠	/	≠	≠	/	–	–	–
HP0061	128	16	125	–	–	–	8	0.5	62.5	≠	≠	/	128	128	1	–	–	–
HP0062	≠	4	31.2	–	–	–	8	0.5	62.5	≠	≠	/	≠	≠	/	–	–	–
Activity spectrum (%)	0	34.4		0	100		6.9	100		0	0		0	0		40	100	

(−): non tested; (/): undetermined; (≠): MIC >128 μg/ml; F: fractional inhibitory concentration × 10^3^; *P* Presence of PAßN, *A* Absence of PAßN, *AMP* Ampicillin, *AMO* Amoxicillin, *MET* Metronidazole, *ERY* Erythromycin, *DOX* Doxycycline, *CLR* Clarithromycin

An increase in potency of 128 and 64-fold was observed with metronidazole in 3.125% (1/32) of the selected MDR isolates, that of clarithromycin was 64 and 40-fold in 40% (2/5) of the selected isolates, 32 to 40-fold with erythromycin in 34.48% (10/29) of the tested isolates and 8-fold increase in potency with doxycycline in 60% (3/5) of the MDR isolates tested (Table 4).

## Discussion

Appropriate selection of antibiotic regimen for treatment of *H. pylori* prior to initiation therapy decreases the exposure to ineffective antibiotics and increases the rate of cure. This highlights the need for susceptibility testing of *H. pylori* isolates prior to the eradication of infection.

In the present investigation, we tested the resistant profile of *H. pylori* isolated from dyspeptic patients attending Gastroenterology Department of two reference health center in Douala-Cameroon, to sixteen routinely used drugs including ampicillin, amoxicillin, co-amoxiclav, penicillin, imipenem, metronidazole, rifabutin, clarithromycin, erythromycin, azithromycin, tetracycline, doxycycline, minocycline, ciprofloxacin, levofloxacin and norfloxacin. Our findings showed that 97.85% (137/140) of the tested *H. pylori* clinical isolates were resistant to metronidazole. Metronidazole is a frequently used drug in Cameroon for other infections like parasitic or genital infections. Hence, the high dose metronidazole use in vivo or abuse of this inexpensive drug may contribute to the increase in metronidazole resistance and therefore it is not unexpected to find such a high level of resistance in our milieu. The use of nitroimidazole for dental infections may also add to selection pressure. The present resistance prevalence is comparable to the 93.2 and 90% resistance rate to MET reported in Africa respectively in Cameroon in 2006 [37] and in Senegal in 2000 [38]. This high prevalence to metronidazole is as might be expected in developing countries. In a Systematic Review reporting data of 31 studies (17 European, 10 Asian, 2 African and 2 American studies) on *H. pylori* antibiotic resistance from 1993 to 2009, the primary metronidazole resistance detected was 92.4% in Africa, 44.1% in America, 37.1% in Asia and 17.0% in Europe [39]; with a statistical significant difference among the four geographic areas. The differences between the resistance rates may reflect the variation in metronidazole usage between continental areas and countries.

Amoxicillin is the only β-lactam used to treat *H. pylori* infection and it is included in most current therapeutic regimens. In the present study, the amoxicillin resistance rate was 97.14%. Our finding is slightly similar to the 85.6% reported by Ndip et al. in 2006 in Cameroon [37]. However the present AMO rate is in contrast with other studies reporting that acquired resistances to AMO are extremely rare. In fact, in Europe, available data from a study found a prevalence rate of 1.1% in Bulgaria [40]; 2.2% in a study enrolling 352 patients in Alaska [41]. Similarly, the prevalence of amoxicillin resistance in Asian countries still low, ranging from 0% in Japan [42]**,** 8.8% in Korea [43] and 1% [44] in Taiwan. Also, in Africa, a study from Senegal enrolling 40 patients reports an absent of amoxicillin resistance [38]. The high rate of resistance in the present study, in comparison with other studies may be due to the use of this drug in a disproportionate manner in our setting.

Three macrolide were used in this study; erythromycin, clarithromycin and azithromycin. Our finding showed a resistance prevalence of 47.85 and 13.57% respectively for erythromycin and clarithromycin. No resistance was detected with azithromycin. Among macrolides, clarithromycin is widely used for *H. pylori* eradication in combination with a proton pump inhibitor with or without a second antibiotic. The present clarithromycin resistance (13.57%) is lower than early clarithromycin resistance prevalence (44.7) detected in Cameroon in 2006 [37], in America (29.3%) [39], in Japan (40.7%) [42], in Italy (36.7%) [45] and in Spain (49.2%) [46]. However, it is comparable to the resistance rate range of 11 to 15% reported in 2 studies performed in Iran [47, 48], in Bulgaria [40], in Denmark [49], in Italy [50], in Korea [43] and in Taiwan [44]. The differences between the resistance rates may reflect the variation in clarithromycin usage between countries. Since high cost of clarithromycin limits the use of this drug in Cameroon, finding such resistant isolates may be partially explained by the primary resistance of *H. pylori* to clarithromycin. Unlike with clarithromycin, the tested isolates did not showed any resistance to azithromycin, suggesting that this later drug could be used instead of clarithromycin. But, this cannot be recommended, since macrolide cross-resistance prevents the use of this entire class of antimicrobials when clarithromycin resistance is present. With regards of the resistance rate to erythromycin, this antibiotics may contribute on the selection of resistant of the tested strains to clarithromycin.

Tetracyclines are currently used in the treatment of *H. pylori* infection as part of quadruple therapy. In this study, resistance rates of 2.86, 5 and 0.71% were detected to tetracycline, doxycycline and minocycline respectively, suggesting that tetracyclines were slightly affected by resistance. The present low resistance rate of the tested clinical isolates to tetracyclines (2.86%) indicates the importance of this drug in eradicating *H. pylori* strains circulating in Cameroon. Tetracyclines are not routinely used in *H. pylori* eradication regimens, therefore finding such a low resistance rate is not unexpected. The present resistance prevalence is in accordance with previous studies reporting that the overall resistance of *H. pylori* to tetracycline is estimated to be around 2% [51]. In fact, the overall tetracycline prevalence rate did not significantly differ between Europe (2.1%), Asia (2.4%) and America (2.7%) [39]. In contrast, increased values were found in Korea (8.8%) [43]; resistance rates up to 20% in Iran (20%) [48] and in Chile (26.8%) [52] and a significant higher resistance of 43.9% in Cameroon [37]. The differences between the resistance rates may reflect the variation in tetracycline usage between our sample populations.

Ciprofloxacin, norfloxacin and levofloxacin were the fluoroquinolones used in this study and their overall resistance prevalence was null. The absence of resistance of the tested clinical isolates to fluoroquinolones indicates the importance of this drug in eradicating *H. pylori* strains circulating in Cameroon. Our results is in accordance with earlier reports revealing that resistance to fluoroquinolones, particularly to levofloxacin was absent in African tested patients [39]. In contrast, the prevalence rate was higher in Europe (24.1%) compared to Asia (11.6%) [39].

No resistance prevalence was detected to rifabutin among the tested isolates. This drug is not routinely used in *H. pylori* eradication regimens, therefore finding no resistance is not unexpected. Moreover, the fact that this drug is used only in a limited number of patients to treat mycobacterial infections may also explain such low resistance rate. Our results is in accordance with some previous studies who did not find resistant among 81 stains tested in Germany [53] in 1999 and 52 strains tested in Japan [54]. However resistance rate of 1.4 and 6.6% were observed respectively among strains isolated from patients in Germany [55] and in England [56].

In the present study, 70% of the tested isolates elicited a multiple drugs resistance pattern. Double resistance with 48.57% was the more frequent, followed by triple (15.71%) and quadruple drugs resistance (5.71%). This multidrug resistance prevalence is higher compared to that obtained in others countries. Torres et al., [57] from Mexico have reported 30.7% double resistance and 8.7% triple resistance among *H. pylori* isolates. Similarly, multiple resistant strains were detected in 21 out of 252 Asiatic patients (8.3%), in 53 out of 352 American patients (15.0%) and in 204 out of 2272 European patients (8.9%) [39]. In general, combined therapy including two antibiotics, and a proton pump inhibitor is used to eradicate *H. pylori* infection as triple therapy. As clarithromycin, metronidazole and amoxicillin are the antibiotics most frequently used, it was interesting to see if both resistances were evenly distributed. A high resistance rate of 42.14% was detected to both metronidazole and amoxicillin among the tested isolates. Since 42.14% (59/140) of the tested *H. pylori* isolates were double resistance to metronidazole and amoxicillin, recurrence *H. pylori* infection could be expected among participants receiving this combined antibiotics as therapeutic regimen. In contrast, the tested isolates elicited a resistance rate less than 5% (4.28%) to both metronidazole and clarithromycin, 1.42% to both metronidazole and tetracycline and 0.71% to both amoxicillin and clarithromycin. Regarding the low resistance rate of these later combined drugs, one could suggest amoxicillin/clarithromycin, metronidazole/clarithromycin, metronidazole/tetracycline with a proton pump-inhibitor as combined therapy for the treatment of *H. pylori* infection in our population, but considering the high resistance rate detected to amoxicillin and metronidazole (97.14 and 97.85% respectively), this cannot be proposed. Combined therapy of fluoroquinolones and tetracyclines could be used as possible agents to achieve a better eradication rate of *H. pylori* infection in these patients with regards to the absence of resistance of the tested *H. pylori* isolates to fluoroquinolones (ciprofloxacin, norfloxacin and levofloxacin) and their low resistance rate to tetracyclines (tetracycline, doxycycline and minocycline).

Single and double resistance rate to both amoxicillin and metronidazole were all high among the tested *H. pylori* clinical isolates. Earlier studies have shown that amoxicillin resistance is mediated by a variety of different mechanisms including mutations in penicillin binding proteins, decreased permeability for the antibiotic [58, 59]. Upon entering the bacterium, metronidazole is reduced to an active anion radical [60]. This forms the active compound, which acts by causing lethal damage to vital molecules such as DNA, RNA, proteins and fatty acids. An extremely low redox potential is required to allow conversion of the drug into the active form, and the cells of the host and most aerobic bacteria lack such a low redox potential [61]. Resistance to metronidazole is mediated by mutations in the rdxA gene in the majority of cases [62], leading to inactivation of the bacterial enzymes needed to activate this antibiotic [62, 63]. Moreover, frxA also been identified in resistant *H. pylori* strains. Thus, mutations in penicillin binding proteins and mutations in the rdxA and frxA genes may be responsible for the high resistance rate detected to amoxicillin and metronidazole respectively among the tested isolates, however these need to be further clarify.

Given the fact that only health centers in the most popular region of the country with high promiscuity, poor sanitation and household hygiene (factors that favour the spread of *H. pylori*) were included, extending the study sites in other region of Cameroon will improve geographic and demographic representation. Moreover, enrolling study participants in community practices will provide data on resistance rates of *H. pylori* isolates from infected persons not treated at medical centers, a population in which resistance rates in *H. pylori* are unknown and may differ significantly from the current study population. Our team are planning to take up these features.

The susceptibility of the selected MDR *H. pylori* isolates to antibiotics in the absence and presence of PAβN showed that this efflux pumps inhibitor have improved the activity of metronidazole, doxycycline, clarithromycin and erythromycin. In fact, in the presence of PAβN, we notice a multi-fold increase in potency and in the spectrum of activity of the above antibiotics (Table 4). This observation suggested that the observed resistance pattern can be blocked by the efflux pump inhibitors that restore the intracellular concentration as well as the activities of the antibiotics [64]. Thus, overexpression of efflux pump may be the partial causative mechanism involved in the multi-drug resistance pattern elicited by the selected MDR isolates against metronidazole, doxycycline, clarithromycin and erythromycin. However others causative mechanism such as inactivation of the antibiotics or structural modification of antibiotic receptors may be also involved and could justified the non-reversibility in the activity of these antibiotics against some tested isolates. The activity spectrum of amoxicillin and ampicillin was not affected in the presence of inhibitors, suggested that efflux pump could not be as the causative mechanism of the observed resistance. In fact, the said drugs as β lactam antibiotics exert their effect by disrupting the manufacture of peptidoglycan, which is main stress-bearing network in the bacterial cell wall. The disruption can occur by blocking either the construction of the subunits of the peptidoglycan or by preventing their incorporation into the existing network. Since these antibiotics acts at the level of bacterial cell wall, they could not be expel from the cell by efflux pumps mechanism.

## Conclusion

Single and double resistance rate to both amoxicillin and metronidazole were all high among the tested *H. pylori* clinical isolates, suggesting that these drugs should be avoided as components of eradication regimen in our sample population. Considering the absence of resistance of the tested *H. pylori* isolates to ciprofloxacin, norfloxacin and levofloxacin and their low resistance rate to antibiotics from tetracycline’s group, we suggest combined therapy of fluoroquinolones and tetracyclines and a proton pump inhibitor as an optimal eradication regimen for the first-line eradication of *H. pylori* infection in our milieu. Also, the administration of metronidazole, doxycycline, clarithromycin and erythromycin in combination with safety efflux pump inhibitor could be better in the management of this infection in our milieu. Ongoing, prospective surveillance of *H. pylori* resistance is essential to ensure that appropriate data are available to guide the choice of therapy, particularly in our context of high-endemicity. Our further attention is focused on the genomic identification of several antibiotics resistance gene among resistant *H. pylori* clinical isolates as an approach to the surveillance of *H. pylori* resistance in Cameroon.

## Data Availability

The datasets used and/or analyzed during the current study are available from the corresponding author on reasonable request.

## Abbreviations

AMC: Co-Amoxiclav
AMO: Amoxicillin
AMP: Ampicillin
AZT: Azithromycin
BHI: Brain Heart Infusion
CIP: Ciprofloxacin
CLR: Clarithromycin
DOX: Doxycycline
ERY: Erythromycin
EUCAST: European Committee on Antimicrobial Susceptibility Testing
FIC: Fractional inhibitory concentration
*H. pylori*: *Helicobacter pylori*
IMP: Imipenem
LEV: Levofloxacin
MDR: Multi-drug resistance
MET: Metronidazole
MIC: Minimal inhibitory concentration
MIN: Minocycline
NOR: Norfloxacin
PAβN: Phe-Arg-naphthylamide
PEN: Penicillin
RIF: Rifabutin
RND: Resistance-nodulation-cell division
TET: Tetracycline
